# Identifying potential DNA methylation markers for the detection of esophageal cancer in plasma

**DOI:** 10.3389/fgene.2023.1222617

**Published:** 2023-10-04

**Authors:** Bing Pei, Guodong Zhao, Zhixin Geng, Yue Wang, Menglin Wang, Xiaomei Wang, Shangmin Xiong, Minxue Zheng

**Affiliations:** ^1^ The Suqian Clinical College of Xuzhou Medical University, Suqian, China; ^2^ Department of Clinical Laboratory, The Affiliated Suqian First People’s Hospital of Nanjing Medical University, Suqian, China; ^3^ Zhejiang University Kunshan Biotechnology Laboratory, Zhejiang University Kunshan Innovation Institute, Kunshan, China; ^4^ Department of R&D, Suzhou VersaBio Technologies Co Ltd., Kunshan, China; ^5^ Suzhou Institute of Biomedical Engineering and Technology, Chinese Academy of Sciences, Suzhou, China

**Keywords:** esophageal cancer, DNA methylation, non-invasive, early detection, plasma

## Abstract

**Background:** Esophageal cancer (EC) is a leading cause of cancer-related deaths in China, with the 5-year survival rate reaching less than 30%, because most cases were diagnosed and treated at the advanced stage. However, there is still a lack of low-cost, efficient, and accurate non-invasive methods for the early detection of EC at present.

**Methods:** A total of 48 EC plasma and 101 control plasma samples were collected in a training cohort from 1 January 2021 to 31 December 2021, and seven cancer-related DNA methylation markers (*ELMO1*, *ZNF582*, *FAM19A4*, *PAX1*, *C13orf18*, *JAM3* and *TERT*) were tested in these samples to select potential markers. In total, 20 EC, 10 gastric cancer (GC), 10 colorectal cancer (CRC), and 20 control plasma samples were collected in a validation cohort to evaluate the two-gene panel.

**Results:** *ZNF582*, *FAM19A4*, *JAM3*, or *TERT* methylation in plasma was shown to significantly distinguish EC and control subjects (*p* < 0.05), and the combination of *ZNF582* and *FAM19A4* methylation was the two-gene panel that exhibited the best performance for the detection of EC with 60.4% sensitivity (95% CI: 45.3%–73.9%) and 83.2% specificity (95% CI: 74.1%–89.6%) in the training cohort. The performance of this two-gene panel showed no significant difference between different age and gender groups. When the two-gene panel was combined with CEA, the sensitivity for EC detection was further improved to 71.1%. In the validation cohort, the sensitivity of the two-gene panel for detecting EC, GC, and CRC was 60.0%, 30.0%, and 30.0%, respectively, with a specificity of 90.0%.

**Conclusion:** The identified methylation marker panel provided a potential non-invasive strategy for EC detection, but further validation should be performed in more clinical centers.

## 1 Introduction

Esophageal cancer (EC) is one of the most common malignant tumors worldwide, with approximately 604,100 new cases and 544,076 deaths in 2020 (Sung et al., 2021). In China, EC ranked fifth in incidence and fourth in mortality among all cancer types according to the latest nationwide statistics (Zheng et al., 2022). China is the country with the heaviest burden of EC in the world. The data from the World Health Organization indicated that EC led to approximately 324,000 new cases and 301,000 deaths in China in 2020, accounting for 53.70% and 55.35% of the global incidence and mortality of EC, respectively (He et al., 2022). Although the incidence rate and 5-year survival rate have showed a trend of gradual improvement in the last decade (Li et al., 2021; He et al., 2022), the 5-year survival rate still remained below 30% (He et al., 2020; Salta et al., 2020) likely because most EC cases were diagnosed and treated at the advanced stage (Early Diagnosis and Treatment Group of the Chinese Medical Association Oncology Branch, 2022). However, if EC can be diagnosed at an early stage, the 5-year survival rate can reach approximately 90% (Early Diagnosis and Treatment Group of the Chinese Medical Association Oncology Branch, 2022). Currently, the gold standard for EC screening is white light endoscopy, which is limited due to its high cost and invasiveness with a low acceptance rate in the Chinese population (Li et al., 2021). Therefore, a new, accurate, and non-invasive early detection method is urgently needed to reduce the risk of EC.

Blood is a widely used clinical sample type, which is easy to collect and process with high throughput. Numerous blood-based new biomarkers for early cancer detection are being studied, and some of them have been applied in clinical practice (Rosado et al., 2019). However, the traditional blood tumor markers, such as CEA and SCC-Ag, lack sufficient sensitivity and specificity, due to which their clinical application has been incompetent (Miyoshi et al., 2022). DNA methylation is an epigenetic modification associated with cell proliferation, apoptosis, and differentiation (Kulis and Esteller, 2010; Pan et al., 2018), and the changes in DNA methylation in cancer have been extensively studied and successfully developed as a powerful diagnostic tool for the early detection of cancer (Koch et al., 2018). Compared with other biomarkers, DNA methylation is a more stable and specific biomarker in blood (Chen et al., 2021; Jamshidi et al., 2022), which can significantly improve the sensitivity in early-stage cancer if multiple DNA methylation markers combined together (Cao et al., 2020; Chen et al., 2021). Our group has also developed several blood-based DNA methylation tests for the early detection of colorectal cancer (Zhao et al., 2019; Zhao et al., 2020) and gastric cancer (Miao et al., 2020), suggesting that blood-based DNA methylation markers may also be applied for the early detection of EC.

In this study, we selected seven cancer-related DNA methylation markers: *engulfment and cell motility 1* (*ELMO1*), *zinc finger protein 582* (*ZNF582*), *family with sequence similarity 19 member A4* (*FAM19A4*), *paired box* *1* (*PAX1*), *chromosome 13 open reading frame 18* (*C13orf18*), *junctional adhesion molecule 3* (*JAM3*), and *telomerase reverse transcriptase* (*TERT*). Among them, *ZNF582*, *PAX1*, and *ELMO1* have been found highly methylated in EC tissues but lack validation in plasma samples (Hu et al., 2017; Qin et al., 2019), while the methylation of *FAM19A4*, *C13orf18*, *JAM3*, and *TERT* has been reported to be associated with squamous cell carcinoma (De Strooper et al., 2016). As 90% of EC cases are esophageal squamous cell carcinoma (Henry et al., 2014), these squamous cell carcinoma-related markers have the potential to serve as early diagnostic markers for EC. Similarly, the performance of most of these markers in the plasma of EC patients has not been reported yet. Based on these principles, we evaluated their performance for EC detection in plasma samples to identify the combination of DNA methylation markers showing the best performance.

## 2 Materials and methods

### 2.1 Sample collection

From 1 January 2021 to 31 December 2021, 59 EC plasma and 102 control plasma samples were enrolled in the training cohort, and from 1 January 2023 to 31 August 2023, 20 EC, 10 gastric cancer (GC), 10 colorectal cancer (CRC), and 20 control plasma samples were collected in the validation cohort. All participants were examined via endoscopy. EC patients were confirmed by pathological diagnoses, and the control subjects were people who underwent physical examination with no evidence of disease. The inclusion criteria for all participants were as follows: patients with the age of 18 years and above, no history of esophageal cancer, no pregnancy, having undergone complete endoscopy, and the results of EC patients were confirmed via pathology. The exclusion criteria for all samples were as follows: hemolysis, insufficient plasma volume, and insufficient cell-free DNA determined using an internal control (*ACTB*). The blood sample was collected from each subject using a 4-mL K_2_EDTA tube and stored at room temperature (20°C ± 5°C) for no more than 24 h. The blood sample was centrifuged two times at 1,350 ± 100 g to separate plasma, and the obtained plasma was stored at −80°C for long-term storage. Finally, 11 EC plasma and one control plasma samples were excluded according to the aforementioned criteria, and 48 EC and 101 control subjects were included for further analysis.

### 2.2 cfDNA extraction and bisulfite treatment

Cell-free DNA (cfDNA) from 0.5–1.0 mL plasma of EC patients and control subjects was purified and bisulfite-treated with Blood Sample Pretreatment Kit for DNA Methylation Detection (Suzhou VersaBio Technologies Co., Ltd., Kunshan, China). The purification and conversion steps were performed according to Miao et al. (2020). Briefly, 1.0 mL lysis buffer and 20 μL of protease K solution were added to each plasma sample and incubated at room temperature for 10 min. Subsequently, 750 μL of ethanol and 20 μL of magnetic bead solution were added to each sample and allowed to incubate at room temperature for 25 min. Following this, 100 μL of elution buffer was introduced to obtain the cfDNA solution. Afterward, 100 μL cfDNA solution, 150 μL of conversion buffer, and 25 μL of protectant were added, and the mixture was incubated at 80°C for 45 min. Then, 1 mL of wash buffer A and 20 μL of magnetic beads were added, followed by a 25-min incubation period. After washing two times in wash buffer B, the purified and converted product was eluted in 100 μL of elution buffer.

### 2.3 Quantitative methylation-specific PCR

The methylation of *ELMO1*, *ZNF582*, *FAM19A4*, *PAX1*, *C13orf18*, *JAM3*, and *TERT* in plasma was analyzed using quantitative methylation-specific PCR (qMSP) assay kits from Suzhou VersaBio Technologies Co., Ltd. (Kunshan, China). The qMSP assay for each methylation marker contained two methylation-specific primers and one methylation-specific probe. The qMSP reaction was performed in a total reaction volume of 30 μL, including 15 μL DNA and 15 μL pre-master mix on an ABI 7500 instrument (Applied Biosystems, Foster City, CA, United States). The concentration of each primer was 0.4 μM, and the concentration of each probe was 0.2 μM. The qMSP reaction was initially denatured at 95°C for 20–30 min, followed by 50 cycles at 95°C for 10 s, 56°C–58°C for 30 s, and 72°C for 0–15 s, and a final cooling to 40°C for 30 s. For each qMSP reaction, an internal control, *ACTB*, was simultaneously detected in the same tube to monitor the reaction and normalize the methylation level of each marker. Positive and negative controls were run in parallel with samples each time. The sequences of primers and probes used in this study are listed in Supplementary Table S1. Samples were analyzed using Applied Biosystems Real-Time PCR Software v2.4.

### 2.4 Blood CEA level measurement

CEA levels were measured using a Roche Cobas 8000 electrochemiluminescence instrument at the Department of Clinical Laboratory of The Affiliated Suqian First People’s Hospital of Nanjing Medical University. The normal reference value was CEA≤5 ng/mL.

### 2.5 Data analysis

The cfDNA concentration was considered insufficient if the Ct value of *ACTB* was more than 40.0; thus, the reaction was considered “valid.” Receiver operating characteristic (ROC) curves were plotted with Ct values or ∆Ct values, and the area under the curve (AUC) values were calculated. Ct values were set to the maximal PCR cycle numbers of 50 for samples with no amplification signals in the qMSP reaction (Supplementary Tables S2, S3) (He et al., 2020). A ∆Ct value was defined as the difference between the Ct values of the methylation marker and *ACTB*. A multinomial logistic regression was performed to calculate the probability, which was used as the test variable to run an ROC curve for two or three biomarker combination. GraphPad Prism 8.0 was used for all statistical analyses, Pearson’s chi-squared test for sensitivity comparison among groups, and the Mann–Whitney U-test for the differences in methylation levels.

## 3 Results

This study included 48 qualified EC samples and 101 qualified control subjects in the training cohort for data analysis (Table 1). The age of EC patients ranged from 53 to 85 years, with a mean of 69.2. The percentage of male patients was 77.1% in the EC group. In the control group, the age of the subjects ranged from 28 to 90 years, with a mean of 59.6, and 41.6% were males (Table 1). In the validation cohort, the mean age for EC, GC, CRC, and control subjects was 71.7, 63.4, 66.7, and 31.3, respectively. The proportion of male patients for EC, GC, CRC, and control subjects was 65.0%, 70.0%, 70.0, and 25.0%, respectively (Table 1).

**TABLE 1:** Demographic information of the subjects enrolled in this study.

	Training cohort		Validation cohort			
	EC	Control	EC	GC	CRC	Control
Total number	48	101	20	10	10	20
Age						
Mean	69.2	59.6	71.7	63.4	66.7	31.3
Range (min–max)	53–85	28–90	53–85	32–83	46–74	23–59
Gender						
Male (n/[%])	37 (77.1)	42 (41.6)	13 (65.0)	7 (70.0)	7 (70.0)	5 (25.0)
Female (n/[%])	11 (22.9)	59 (58.4)	7 (35.0)	3 (30.0)	3 (30.0)	15 (75.0)

To identify potential DNA methylation markers for EC, the Ct values and △Ct values for each marker were used to plot ROC curves, and potential markers were selected based on AUC values and *p*-values. As shown in Table 2, for ROC curves plotted with Ct values in the training cohort, only *ZNF582* could significantly discriminate EC from control subjects, with an AUC value of 0.660. If ROC curves were plotted with △Ct values, *ZNF582*, *FAM19A4*, *JAM3*, and *TERT* all showed a significant difference between EC and control subjects, and all of their AUC values were greater than or equal to 0.600. Particularly for *ZNF582*, the AUC value obtained with △Ct values was larger than that obtained with Ct values.

**TABLE 2:** Discrimination of each DNA methylation marker in the training cohort for esophageal cancer detection in plasma samples.

DNA methylation marker	Ct value		△Ct value	
	AUC	*p*-value	AUC	*p*-value
ELMO1	0.502	0.963	0.58	0.129
ZNF582^a^	0.660	0.003	0.677	0.001
FAM19A4^a^	0.544	0.371	0.600	0.043
PAX1	0.568	0.197	0.552	0.334
C13orf18	0.528	0.55	0.512	0.815
JAM3^a^	0.512	0.734	0.611	0.029
TERT^a^	0.587	0.051	0.662	0.001

^a^ Markers advanced to further analysis.

**FIGURE 1:**
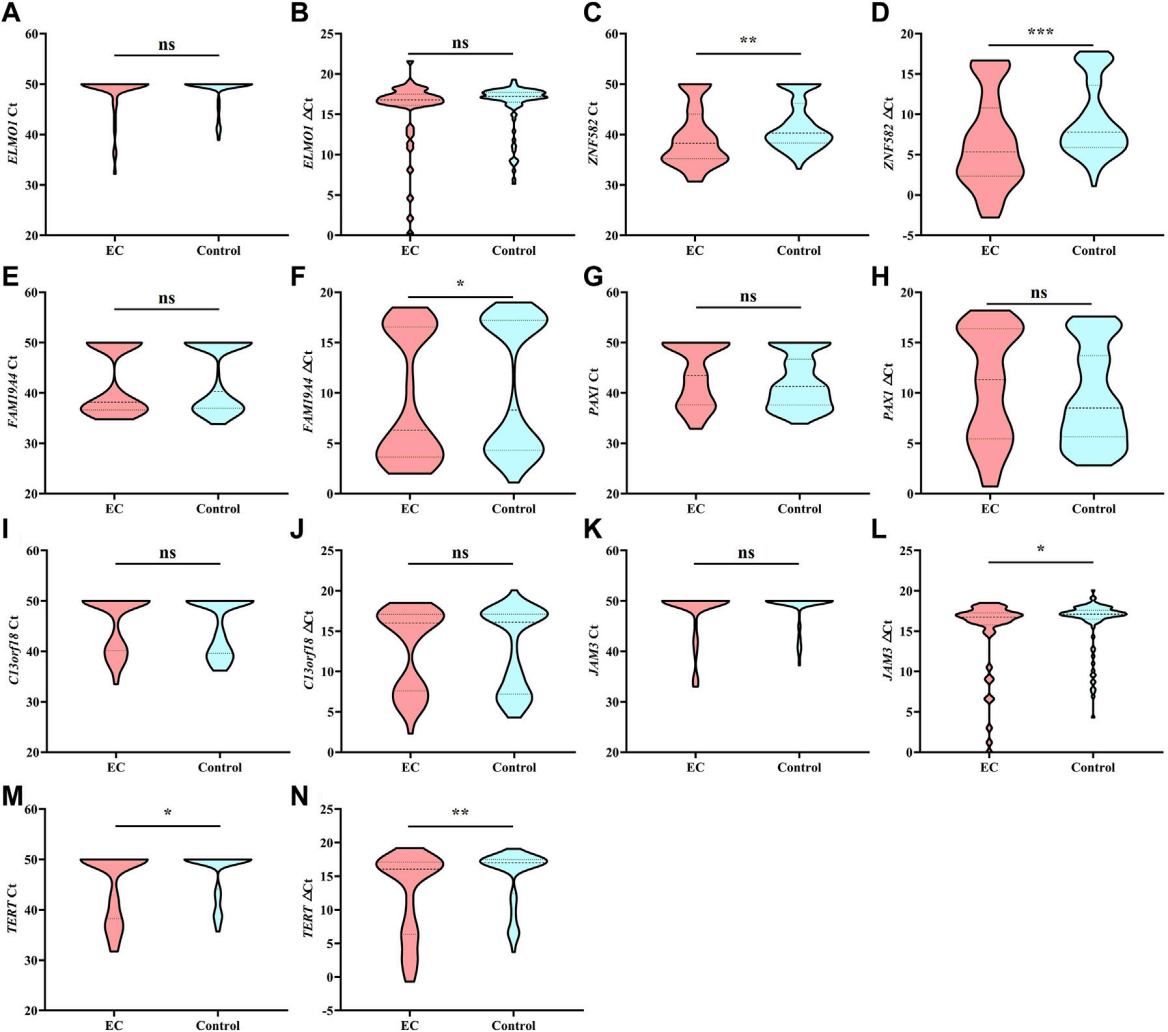
Methylation level of each DNA methylation marker in the training cohort analyzed by Ct values and △Ct values. *, *p* < 0.05; **, *p* < 0.01; ***, *p* < 0.001; ns, no significant difference.

Each DNA methylation marker was further evaluated for its ability to discriminate between EC and control plasma samples in the training cohort with both Ct and △Ct values as they represent absolute and normalized methylation levels, respectively. Only the Ct values of *ZNF582* and *TERT* showed a significant difference between the EC and control groups (Figures 1C,M). In comparison, the △Ct values of *ZNF582*, *FAM19A4*, *JAM3*, and *TERT* displayed significant differences between the EC and control groups (Figures 1D,F,L,M). Comprehensive analysis of the aforementioned results showed the same trend between AUC values and methylation levels. Therefore, the Ct value of *ZNF582* and △Ct values of *ZNF582*, *FAM19A4*, *JAM3*, and *TERT* were chosen for combination biomarker analysis.

**FIGURE 2:**
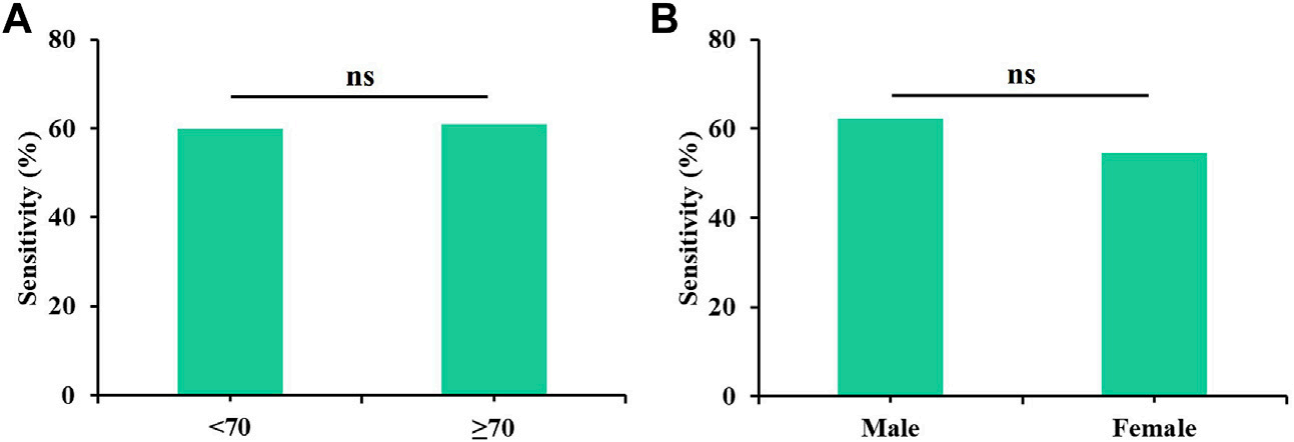
Sensitivities of the two-gene panel containing *ZNF582* and *FAM19A4* in the training cohort for the detection of esophageal cancer in different groups. **(A)** age, **(B)** gender.

All possible combinations of multiple methylation markers in the training cohort are displayed in Table 3, and their AUC values and *p*-values were calculated. The combination of △Ct values for *ZNF582* and *FAM19A4* showed the largest AUC value. Meanwhile, the △Ct value of *ZNF582* alone showed a sensitivity of 43.8% (95% CI: 29.8%–58.7%), with a specificity of 93.1% (95% CI: 85.6%–96.9%), and the sensitivity and specificity of *FAM19A4* △Ct value alone were 25.0% (95% CI: 14.1%–39.9%) and 90.1% (95% CI: 82.1%–94.9%), respectively. When *ZNF582* and *FAM19A4* were combined, the sensitivity was improved to 60.4% (95% CI: 45.3%–73.9%), while the specificity decreased to 83.2% (95% CI: 74.1%–89.6%). Nonetheless, the combination of *ZNF582* and *FAM19A4* still achieved the largest Youden index compared to single markers (Table 4). As for the positive predictive value (PPV), the combination of *ZNF582* and *FAM19A4* also achieved a compromise result compared with the single gene; however, it has the highest negative predictive value (NPV), which represents that the assay can detect more EC cases with higher sensitivity (Table 4). Therefore, the combination of *ZNF582* and *FAM19A4* △Ct values was the most discriminant two-gene panel for the detection of EC. Sensitivities of this two-gene panel showed no significant difference between different age and gender groups (Figure 2). Based on the aforementioned results, the cut-off values for this two-gene panel are as follows: *ZNF582* △Ct < 4.30, *FAM19A4* △Ct < 3.65.

**TABLE 3:** Discrimination of several methylation marker combinations in the training cohort for esophageal cancer detection in plasma samples.

Methylation marker combination	AUC	*p*-value
*ZNF582* + △Ct *FAM19A4*	0.661	0.002
△Ct *ZNF582* + △Ct *FAM19A4*	0.675	0.001
*ZNF582* + △Ct *JAM3*	0.648	0.004
△Ct *ZNF582* + △Ct *JAM3*	0.666	0.002
*ZNF582* + △Ct *TERT*	0.666	<0.001
△Ct *ZNF582* + △Ct *TERT*	0.668	<0.001
*ZNF582* + △Ct *FAM19A4* + △Ct *JAM3*	0.641	0.007
△Ct *ZNF582* + △Ct *FAM19A4* + △Ct *JAM3*	0.663	0.001
*ZNF582* + △Ct *FAM19A4* + △Ct *TERT*	0.655	0.001
△Ct *ZNF582* + △Ct *FAM19A4* + △Ct *TERT*	0.663	0.001
*ZNF582* + △Ct *FAM19A4* + △Ct *JAM3* + △Ct *TERT*	0.656	0.001
△Ct *ZNF582* + △Ct *FAM19A4* + △Ct *JAM3* + △Ct *TERT*	0.666	0.001

**TABLE 4:** Sensitivities and specificities of potential DNA methylation markers for the detection of esophageal cancer in the training cohort.

	Sensitivity (95% CI, %)	Specificity (95% CI, %)	Youden index (%)	PPV (%)	NPV (%)
△Ct *ZNF582*	43.8 (29.8–58.7)	93.1 (85.6–96.9)	36.9	75.0	77.7
△Ct *FAM19A4*	25.0 (14.1–39.9)	90.1 (82.1–94.9)	15.1	54.5	71.6
△Ct *ZNF582* + △Ct *FAM19A4*	60.4 (45.3–73.9)	83.2 (74.1–89.6)	43.6	63.0	81.5

PPV, positive predictive value; NPV, negative predictive value.

**FIGURE 3:**
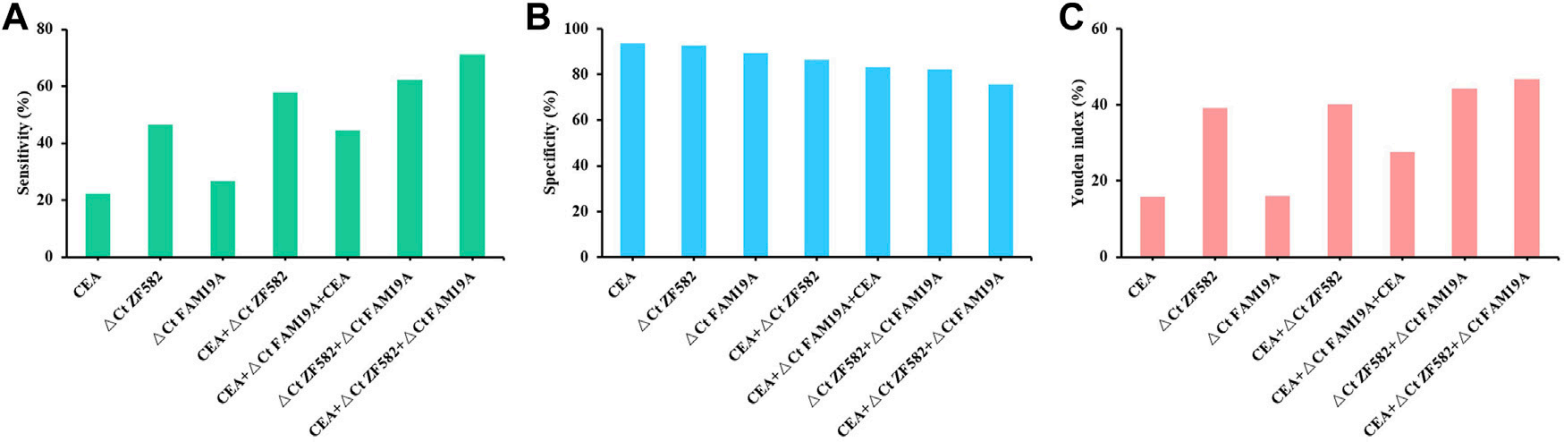
Sensitivity **(A)**, specificity **(B)**, and Youden index **(C)** of methylation markers combined with CEA for the detection of esophageal cancer.

**FIGURE 4:**
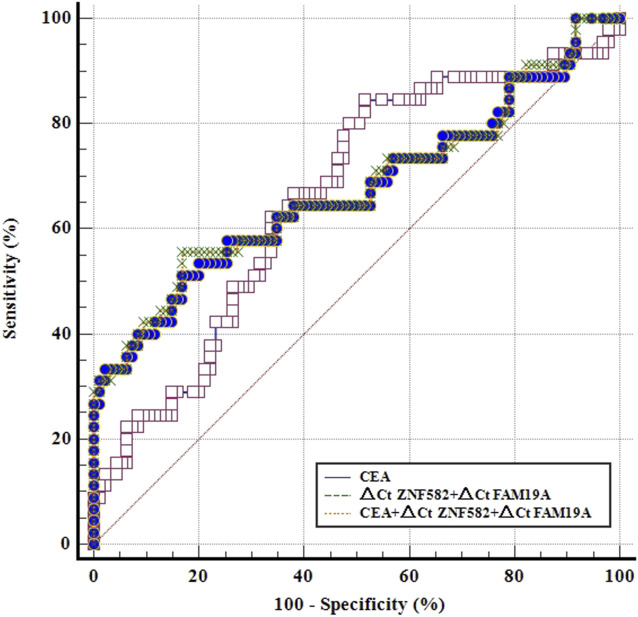
ROC curves for methylation markers combined with CEA for the detection of esophageal cancer in the training cohort.

CEA is a commonly used blood-based protein marker for cancer detection in clinics. The diagnostic efficacies of CEA and two-gene DNA methylation panel for EC detection were compared. In 45 EC patients, the sensitivity of CEA was only 22.2%, which was less than the sensitivities of *ZNF582* and *FAM19A4* (46.7% and 26.7%, respectively) (Figure 3A). In contrast, CEA showed the highest specificity of 93.7% among the three markers (Figure 3B). When *ZNF582* or *FAM19A4* was combined with CEA, their sensitivities increased to 57.8% and 44.4% (Figure 3A), while their specificities were 86.3% and 83.2%, respectively (Figure 3B). However, if the two-gene panel was combined with CEA, sensitivity was improved from 62.2% to 71.1% and specificity decreased from 83.2% to 75.8%. When Youden indexes were compared, the combination of two-gene panel and CEA showed the largest Youden index of 46.9%, whereas Youden indexes of two-gene panel and CEA alone were 43.6% and 15.9%, respectively (Figure 3C). The AUC value of CEA for discriminating EC from control subjects was 0.661 (95% CI: 0.563–0.759), and the AUC value of two-gene panel was 0.673 (95% CI: 0.566–0.779). In comparison, the combination of two-gene panel and CEA improved the AUC value to 0.677 (95% CI: 0.571–0.784) (Figure 4).

**FIGURE 5:**
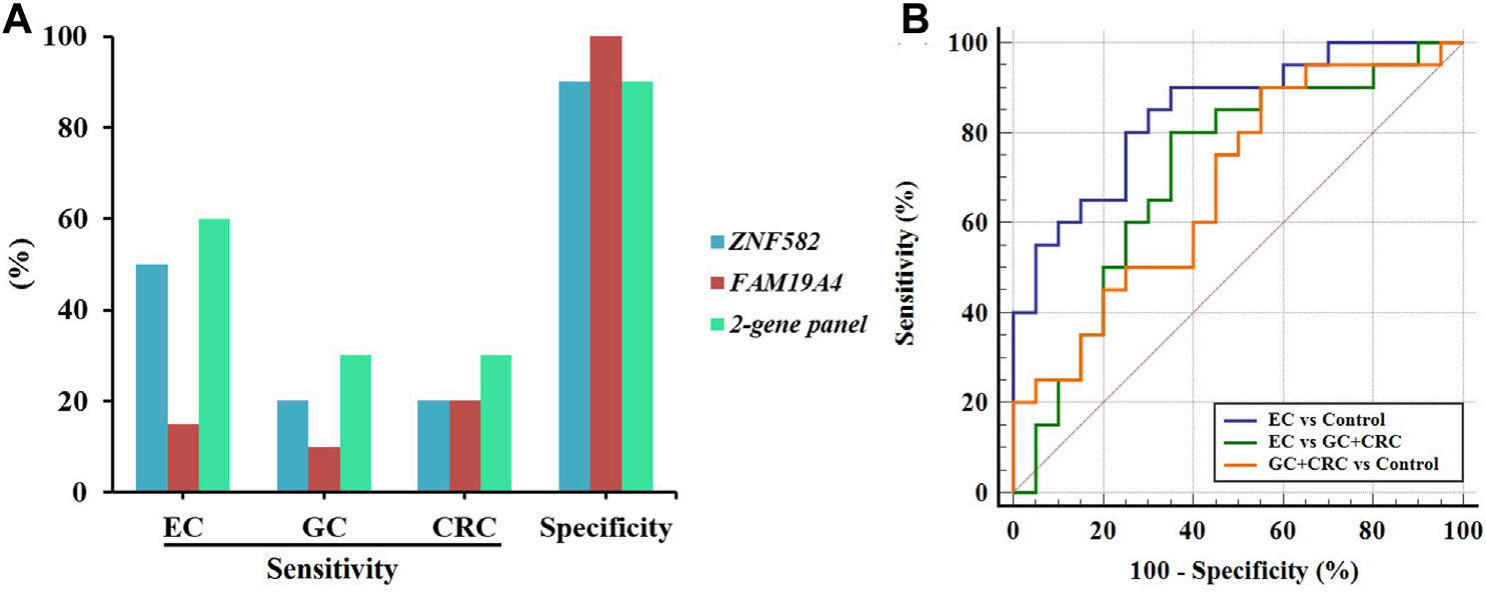
Performance of the two-gene panel for the detection of EC in the validation cohort. **(A)** Sensitivities and specificities of the two-gene panel for the detection of EC, GC, and CRC. **(B)** Comparison of ROC curves of the two-gene panel for the detection of EC, GC, and CRC.

In order to further evaluate the performance of the two-gene panel for the detection of EC, we included 20 EC, 10 GC, 10 CRC, and 20 control subjects in a validation cohort and compared its sensitivity and specificity in each group. As shown in Figure 5A, the two-gene panel displayed higher sensitivity (60.0%) than single genes (50.0% and 15.0%) for the detection of EC, with a specificity of 90.0%. In contrast, the sensitivity of the two-gene panel in detecting GC and CRC was only 30.0%, demonstrating its higher specificity for EC. Additionally, as shown in Figure 5B, the two-gene panel exhibited an AUC value of 0.845 when distinguishing EC from control subjects. The AUC value for distinguishing EC from the combination of GC and CRC is 0.710, while the AUC value for distinguishing the combination of GC and CRC from control subjects was 0.682.

## 4 Discussion

EC is a leading cause of cancer-related deaths in China, and early detection and screening is one of the most effective strategies for reducing its incidence and mortality. DNA methylation is the most reliable biomarker at present, with high stability and specificity, and several blood-, stool-, or cell-based commercial kits have been developed and approved by regulatory authorities for the early detection of colorectal cancer, lung cancer, and cervical cancer (Potter et al., 2014; Zhang et al., 2017; Dippmann et al., 2020; Wang et al., 2022). These positive outcomes suggested that DNA methylation markers might be developed into non-invasive diagnostic methods for the early detection of EC.

In this study, we tested the methylation of *ELMO1*, *ZNF582*, *FAM19A4*, *PAX1*, *C13orf18*, *JAM3*, and *TERT* in plasma for their abilities to discriminate EC from control subjects. The results of *ELMO1*, *PAX1*, and *C13orf18* methylation showed no significant difference between the EC and control groups. In Tang et al. (2019), *PAX1* methylation detected EC with 96.0% sensitivity and 51.4% specificity in tissue samples, and Qin et al. (2019) showed that *ELMO1* methylation significantly discriminated EC from control subjects in tissue and plasma samples. The inconsistency between our results and the previous studies may be due to the following reasons: a) the quantity of methylated genomic DNA in tissue samples is far more than that in a small volume of plasma; thus, *PAX1* methylation in plasma may not be detected due to low cfDNA amount. b) The plasma volume used in this study is only approximately 1/4 of that used in the previous study, which may have affected *ELMO1* sensitivity, as our previous study demonstrated that decreasing plasma volume significantly decreased sensitivities of methylation markers in plasma (Zhang et al., 2021).

The methylation of *FAM19A4*, *JAM3*, and *TERT* has been identified as diagnostic biomarkers for cervical neoplasia (Jiang et al., 2012; Yin et al., 2015; Bonde et al., 2021). Cervical cancer includes two histologic subtypes, cervical squamous cell carcinoma and cervical adenocarcinoma (Kang et al., 2006), while EC includes the same two histologic subtypes, esophageal adenocarcinoma and esophageal squamous carcinoma (Testa et al., 2017). Therefore, cervical cancer and EC may have similar pathological processes. In addition, several cervical cancer-related methylation markers have also demonstrated aberrant DNA methylation in EC (Rad et al., 2016). *ZNF582* methylation is another confirmed marker associated with cervical cancer (Li et al., 2019), and *ZNF582* methylation has also been reported as a potential marker for EC detection in tissue samples (Tang et al., 2019), although the study of *ZNF582* methylation in plasma samples is insufficient. After three rounds of selection, *ZNF582* and *FAM19A4* methylation markers were found to be the best combination for the detection of EC in plasma. The results in Table 2 and Figure 1 indicated that while *ZNF582* methylation was the best single marker for EC detection in plasma, the combination of *ZNF582* and *FAM19A4* methylation could further improve the diagnostic capability.

CEA is a commonly used traditional cancer marker for cancer treatment monitoring and detection (Leja and Linē, 2021), but its sensitivity for EC detection is no more than 30% such that CEA is always combined with other markers to improve its sensitivity (Bagaria et al., 2013). In this study, CEA showed 22.2% sensitivity and 93.7% specificity for EC detection, while the two-gene panel showed a significantly higher sensitivity of 62.2%, almost 2.8-fold of CEA. Furthermore, if the two-gene panel and CEA were combined, sensitivity further increased to 71.1%. CEA and the two-gene panel are both blood-based markers; thus, only one tube of blood needs to be drawn for the simultaneous detection of these two markers. Therefore, it can not only improve the sensitivity of EC detection but also increase the screening throughput, allowing more people to participate in the early detection of EC.

There are some limitations to this study. The number of EC cases was relatively small, and more subjects need to be enrolled in multiple centers in future studies to validate the performance of the two-gene panel for EC detection. In addition, the detailed pathological information of EC patients, such as cancer stage, tumor size and differentiation, and more characteristics of the patients (smoking, cancer type, and medication), was not collected and analyzed. Such information can help us evaluate the performance of DNA methylation markers more comprehensively.

## 5 Conclusion

In this study, we demonstrated that four methylation markers can significantly distinguish EC from control subjects in plasma, and two methylation markers, *ZNF582* and *FAM19A4*, were selected as the combination with the most potential for the early detection of EC. Therefore, non-invasive detection of EC based on DNA methylation markers in plasma can be an effective strategy to reduce the incidence and mortality of EC.

## Data Availability

The raw data supporting the conclusion of this article will be made available by the authors, without undue reservation.
